# “Seeing Pain Differently”: A Qualitative Investigation Into the Differences and Similarities of Pain and Rheumatology Specialists’ Interpretation of Multidimensional Mobile Health Pain Data From Children and Young People With Juvenile Idiopathic Arthritis

**DOI:** 10.2196/12952

**Published:** 2019-07-02

**Authors:** Rebecca Rachael Lee, Amir Rashid, Daniela Ghio, Wendy Thomson, Lis Cordingley

**Affiliations:** 1 NIHR Manchester Musculoskeletal Biomedical Research Centre Central Manchester University Hospitals NHS Foundation Trust Manchester Academic Health Science Centre Manchester United Kingdom; 2 Arthritis Research UK Centre for Epidemiology, Centre for Musculoskeletal Research Faculty of Biology, Medicine and Health The University of Manchester Manchester United Kingdom; 3 Primary Care and Population Science Faculty of Medicine University of Southampton Southampton United Kingdom; 4 Arthritis Research UK Centre for Genetics and Genomics, Centre for Musculoskeletal Research Faculty of Biology, Medicine and Health The University of Manchester Manchester United Kingdom; 5 Division of Musculoskeletal and Dermatological Sciences Faculty of Biology, Medicine and Health The University of Manchester Manchester United Kingdom

**Keywords:** mHeath, pain assessment, juvenile idiopathic arthritis, focus group, qualitative research

## Abstract

**Background:**

In contrast to the use of traditional unidimensional paper-based scales, a mobile health (mHealth) assessment of pain in children and young people (CYP) with juvenile idiopathic arthritis (JIA) enables comprehensive and complex multidimensional pain data to be captured remotely by individuals. However, how professionals use multidimensional pain data to interpret and synthesize pain reports gathered using mHealth tools is not yet known.

**Objective:**

The aim of this study was to explore the salience and prioritization of different mHealth pain features as interpreted by key stakeholders involved in research and management of pain in CYP with JIA.

**Methods:**

Pain and rheumatology specialists were purposively recruited via professional organizations. Face-to-face focus groups were conducted for each specialist group. Participants were asked to rank order 9 static vignette scenarios created from real patient mHealth multidimensional pain data. These data had been collected by a researcher in a separate study using My Pain Tracker, a valid and acceptable mHealth iPad pain communication tool that collects information about intensity, severity, location, emotion, and pictorial pain qualities. In the focus groups, specialists discussed their decision-making processes behind each rank order in the focus groups. The total group rank ordering of vignette scenarios was calculated. Qualitative data from discussions were analyzed using latent thematic analysis.

**Results:**

A total of 9 pain specialists took part in 1 focus group and 10 rheumatology specialists in another. In pain specialists, the consensus for the highest pain experience (44%) was poorer than their ranking of the lowest pain experiences (55%). Conversely, in rheumatology specialists, the consensus for the highest pain experience (70%) was stronger than their ranking of the lowest pain experience (50%). Pain *intensity* was a high priority for pain specialists, but rheumatology specialists gave high priority to *intensity* and *severity* taken together. Pain *spread* was highly prioritized, with the *number of pain locations* (particular areas or joints) being a high priority for both groups; *radiating pain* was a high priority for pain specialists only. Pain *emotion* was challenging for both groups and was only perceived to be a high priority when specialists had additional confirmatory evidence (such as information about pain interference or clinical observations) to validate the pain emotion report. Pain qualities such as particular word descriptors, use of the color red, and fire symbols were seen to be high priority by both groups in interpretation of CYP pain reports.

**Conclusions:**

Pain interpretation is complex. Findings from this study of specialists’ decision-making processes indicate which aspects of pain are prioritized and weighted more heavily than others by those interpreting mHealth data. Findings are useful for developing electronic graphical summaries which assist specialists in interpreting patient-reported mHealth pain data more efficiently in clinical and research settings.

## Introduction

### Pain in Juvenile Idiopathic Arthritis

It is challenging to assess and manage pain in children and young people (CYP) with juvenile idiopathic arthritis (JIA). A significant proportion of CYP with JIA report severe pain [1,2], and for 17% of CYP, pain remains consistently high throughout long periods of the disease [3]. Pain in the context of JIA is unpredictable, and importantly, fluctuations in pain qualities can act independently of the levels of inflammatory disease processes in these patients [2,4,5]. This means that separate assessments of pain and disease domains are essential. Self-report of pain is advocated as the *gold standard* in assessment [6]; however, for CYP it can be particularly challenging to articulate and summarize their pain [7].

### Pain Assessment and Communication Issues

Pain is an inherently subjective concept [8], hence pain experiences are difficult to communicate to others. Communication between patients with JIA and health care professionals (HCPs) is further complicated by infrequent clinic visits, where pain is reported retrospectively and often by proxies, rather than children themselves [9-11]. Most reporting tools require researchers or HCPs to interpret scores from linear or unidimensional self-reported pain scales [12]. Pain interpretation is also a subjective process, and it can be difficult for others to interpret pain experiences as expressed by CYP [13,14]. The salience of scale points may not be equivalent (eg, a difference between a 7 and 8 on a unidimensional numerical rating scale may hold more significance than that between a 1 and 2) to those considering patients pain scores. In more recent research, there has been a move toward using multidimensional tools that collect even more comprehensive data on several aspects of the pain experience, including location, intensity, severity, emotion, and other pain qualities such as pain interference [15-17]. These tools can provide many advantages for pain assessment, particularly in their digital forms whereby pain can be recorded frequently (ensuring richer pain data collection) and remotely outside of the clinic (avoiding recall bias) [17-19]. Scores from these more comprehensive, multidimensional pain depictions then require synthesis and interpretation.

Methods of interpreting pain assessments are largely dependent on subjective scoring systems, which have been found to be particularly problematic in HCPs managing CYP with JIA. Some studies suggest HCPs provide overestimations of their young patients’ pain [11], and others have found that they are more likely to underestimate [20]. Little is known about the decision-making processes behind how others score and interpret CYP pain and how or why they might report overestimations or underestimations.

### Multidimensional Pain Interpretation

Recent research on the implementation of multidimensional pediatric pain assessment tools has primarily focused on development [21,22] rather than on the utilization and interpretation of information from such measures. Given the increase in the availability and adoption of mHealth tools for the management of chronic pediatric conditions, it is important and necessary to understand how the data collected via these methods are being used to make choices about the management of long-term conditions with associated pain symptomatology [23]. The primary aim of this study was to identify which aspects of pain were considered to be the most salient in the prioritization and utilization of CYP pain data from an mHealth pain assessment tool, by 2 key stakeholder groups involved in the interpretation of pain in JIA: pain specialists and pediatric rheumatology specialists.

## Methods

### Study Design

A total of 2 separate face-to-face semistructured focus groups were undertaken, 1 with pain specialists and 1 with pediatric rheumatology specialists. Stimulus material in the form of real mHealth multidimensional pain data from CYP with JIA was presented to participants in the form of vignette scenarios. This approach was used to provoke and elicit underlying opinions about the degree of pain represented by these datasets and to stimulate a structured discussion within the focus groups.

### Sample and Recruitment

In total, 2 groups of participants recruited through purposive sampling took part in the study: international academic pediatric pain experts (termed pain specialists throughout the paper) and HCPs managing CYP with JIA in pediatric rheumatology departments in the United Kingdom National Health Service (NHS; termed rheumatology specialists throughout the paper). These groups were recruited to reflect 2 key professional stakeholder groups and interests involved in the research and clinical management of pain in CYP with JIA. Participants were selected based upon their specializations in pain assessment and/or management and were members of professional member organizations (The International Association for the Study of Pain and/or The British Society for Paediatric and Adolescent Rheumatology). Selected participants were emailed a participant information sheet with the aims of the study and briefed about what would be involved if they chose to participate. Within the information sheet, participants were also given a brief background to the rationale of the study, the research group’s work, and who the researchers involved in the study were. Interested participants responded directly to the authors and were encouraged to send the email to colleagues with similar interests or specializations (snowball sampling [24]).

### Setting

A total of 2 separate focus groups were conducted, with the pain specialist focus group held at an international pain conference (United States) and the rheumatology specialist focus group held at a national rheumatology conference (United Kingdom).

#### Materials

**Figure 1:**
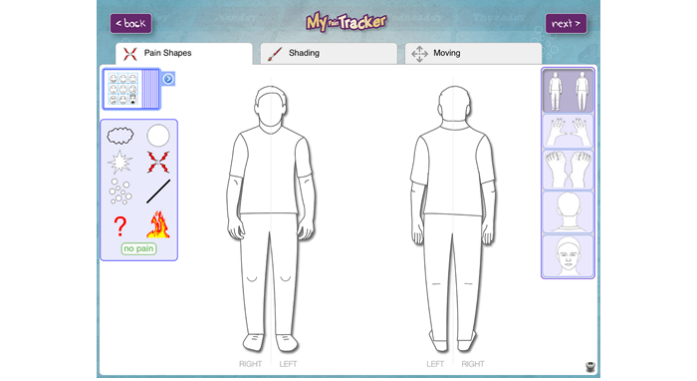
Screenshot of the My Pain Tracker main user page.

This study used real-world mHealth multidimensional pain data collected from CYP with JIA using My Pain Tracker (MPT), an mHealth pain assessment app for iPads (version 1.1.5). MPT has been found to be a usable, valid, and acceptable pain assessment and communication tool for CYP with JIA within the research group, when compared with the use of other scales (Visual Analog Scales and the Faces Pain Scale). MPT was first adapted from an interview tool for reporting pain in CYP in forensic settings [25-26]. Since its initial conception in 2000, the tool has been adapted into appropriate versions for different pain contexts such as acute postoperative pain [27] and recurrent pain (personal communication by J Twynholm, 2009) and in its latest version used in this study, it has been used for the assessment and communication of persistent pain in the specific context of JIA [28]. The tool enables CYP to discuss their musculoskeletal pain symptoms with a researcher or HCP through pain recording features (pain intensity, severity, location/spread, pictorial representations such as symbols and colors, word descriptors, and emotions; see Figure 1 for a screenshot of MPT main user page). Within the app, intensity is depicted through the throb of pain (the speed of movement of the chosen symbol), and severity is signified by the size of the pain. The pain quality symbols within the app were designed by CYP in a study that aimed to capture how they represent painful experiences through drawings [25]. In pilot tests, it has been found that these symbols do not have one unique meaning to CYP, instead, they are used to represent and signify several pain meanings dependent on individual connotation.

Vignette scenarios based upon real MPT data were provided as a sample set of stimuli for discussion. In total, 9 different vignette scenarios were chosen to represent a breadth of different multidimensional self-reports of pain experiences (Multimedia Appendix 1). The vignette scenarios were originally collected from CYP with JIA as part of an acceptability study. In the original study, MPT was completed by CYP (aged between 5 and 16 years) in a semistructured interview with a clinical psychologist trainee or researcher. Original data for the vignettes were collected at a tertiary pediatric rheumatology outpatient clinic in the North-West of England from participants with JIA enrolled in the Childhood Arthritis Prospective study [29].

### Procedure

This paper has been reported in accordance with the Consolidated Criteria for Reporting Qualitative Research, a 32-items checklist [30] (Multimedia Appendix 2). Participant information sheets were provided to selected participants. Email confirmation to attend focus groups provided written consent. In addition to this, verbal consent to participate in the study was obtained at the beginning of the focus groups. Participants attending the focus groups were given a standardized presentation by the study team (AR, DG, and RRL) about the development of MPT, including the app, purpose, format, and completion process. At the time of data collection, AR was a research associate (trained to PhD level in Psychology and Medical research), and DG and RRL were PhD students (trained to MSc level in Health Psychology and trained in Psychology and Medical research). All researchers involved in data collection and analysis had conducted and been involved in previous qualitative research studies and were closely supervised in preparing and conducting focus groups by LC (senior lecturer and practitioner in Health Psychology). The researchers conducting this study did not have any particular experience of managing pain, either in a pain or rheumatology-focused medical context.

Participants were given a demonstration of how MPT works and were given the opportunity to briefly input mHealth data themselves. After the presentation, participants were asked to consider the vignette scenarios and rank them from *highest pain* to *lowest pain*. Participants were then asked to take part in group discussions to explain the reasoning behind their rankings. Focus group discussions provided qualitative data for analysis and were audio-recorded and transcribed verbatim for analysis. All audio-recorded interviews were uploaded to and analyzed in NVivo 10 (QSR International, Doncaster, Australia). Field notes collected by the researchers during the conduct of focus groups were used to provide additional context to the analytical process.

### Data Analysis

Focus group data were analyzed using a step-by-step guide for conducting deductive latent thematic analysis by RRL and LC [31]. Latent thematic analysis is a technique that identifies meaningful patterns within data and involves interpretation of those patterns beyond description. Coders defined what would be considered a significant theme before data analysis (issues about pain prioritization and interpretation of pain features, in line with the aims of the study) and then became familiar with the data by repeatedly reading transcripts and listening to the audio recordings of focus groups. The author then generated initial thoughts and ideas (initial codes) before searching for larger themes that grouped codes together. Major themes and subthemes were reviewed and named. Thematic maps for each major theme were produced to organize data within and between participant accounts. Underlying ideas, assumptions, and beliefs about the prioritization of different pain facets were interpreted. Inter-rater reliability and validation of emerging themes was conducted by RRL and LC, who independently coded and discussed sections of both focus group data [32]. Frequencies for the highest and the lowest pain vignette rankings were calculated from paper-based feedback from participants.

## Results

### Participant Characteristics

A total of 19 participants took part in the focus groups, 9 pain specialists in 1 focus group (participants 1-9) and 10 rheumatology specialists (participants 10-19) in the other focus group (see Table 1 for participants’ professional background and current work contexts). The pain specialist focus group lasted for approximately 48 min, and the rheumatology specialist focus group ran for approximately 75 min.

### Vignette Ranking

#### Highest Pain Vignettes

Both groups selected vignette 6 (named *Ben*, see Multimedia Appendix 1) to represent the individual with the highest pain; 44% (4/9) of the pain specialist participants and 70% (7/10) of the rheumatology specialist group chose this vignette as the worst pain experience.

#### Lowest Pain Vignette

The most commonly chosen lowest pain vignette by pain specialists was vignette 2 (named *Samantha*), with 55% (5/9) voting for this. The vignette ranked the lowest by rheumatology specialists was vignette 3 (named *Anna*), with 50% (5/10) of professionals in this focus group voting for this.

#### Reordering Vignettes

Given the opportunity to rerank vignettes at the end of group discussions, none of the pain specialists chose to rerank any of their choices. However, in the rheumatology specialist group, 50% (5/10) of participants chose to reorder at least 1 of the vignettes they had chosen previously. Rheumatology specialists discussed some of the facets of pain that were prioritized differently in their reinterpretation of pain rankings following group discussions. These included different weightings upon age, emotion, labeling, number of sites, and severity.

### Qualitative Themes of Pain Quality Prioritization

#### Overview

**Figure 2:**
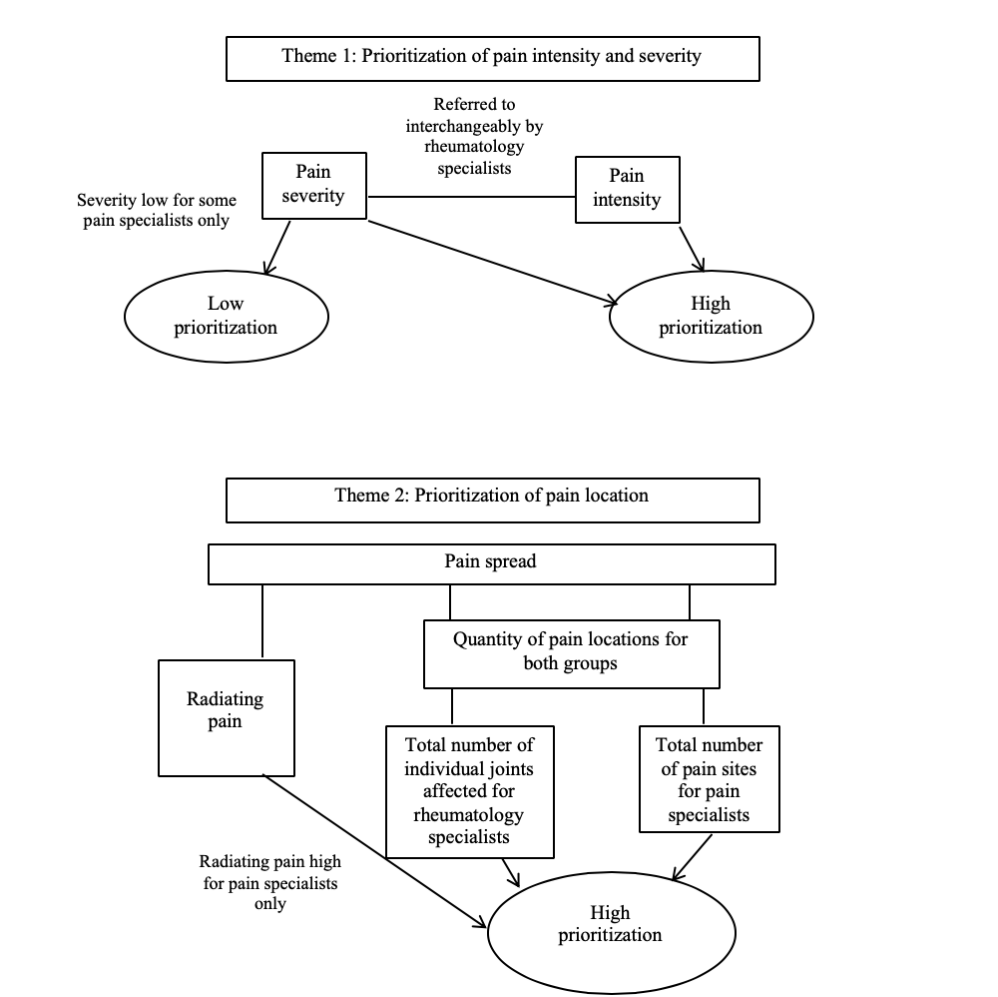
Thematic maps for themes 1 and 2.

**Figure 3:**
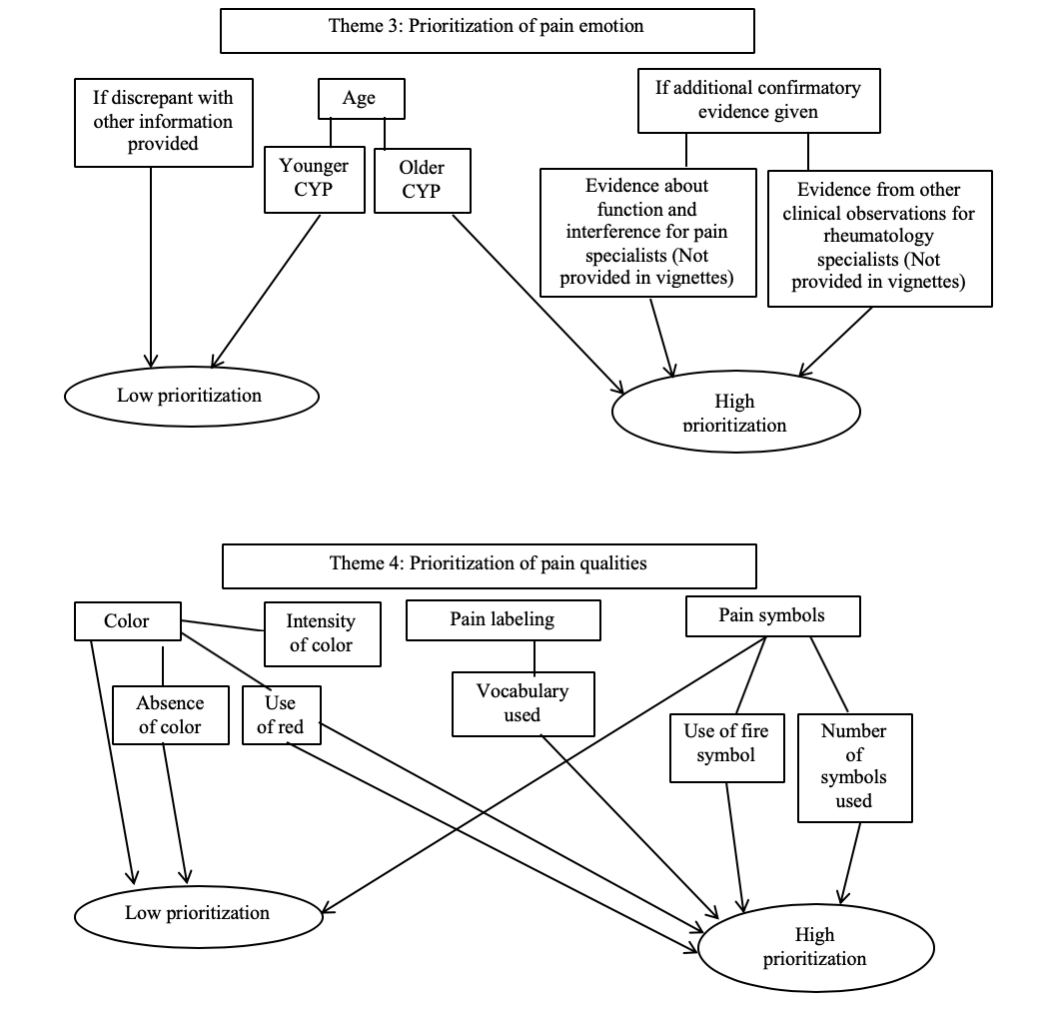
Thematic maps for themes 3 and 4.

In total, 4 major themes were identified throughout the analysis. Themes were deductive in that they were based upon and informed by the specific components of data collected by MPT and included the prioritization of (1) pain intensity and severity, (2) pain location, (3) pain qualities, and (4) pain emotion (see Figures 2 and 3 for thematic maps of data). The thematic maps show the relationship between the key points discussed and divergences between pain and rheumatology specialist groups. Narrative accounts of each theme are presented alongside supporting quotations.

**Table 1:** Professional background of pain specialists and rheumatology specialists.

Professional backgrounds of the specialists and countries they worked in		Frequency (n)
**Pain specialists’ background**		
	Nursing	2
	Health sciences	1
	Psychology	4
	Medicine	1
	Anesthesiology	1
**Country pain specialists worked in**		
	Canada	5
	United States	2
	United Kingdom	2
**Rheumatology specialists’ background**		
	Consultant rheumatologists	3
	Pediatricians (with rheumatology interest)	4
	Nursing	1
	Physiotherapy	1
	Occupational therapy	1
**Country rheumatology specialists worked in**		
	United Kingdom	10

##### Theme 1: Prioritization of Pain Intensity and Severity

The high prioritization of pain intensity was uncontentious for pain specialists. Some talked about the importance of summing intensity information if pain was present in more than 1 area:

I was sort of mentally trying to take the averages of the intensities across the locations.

Pain specialist 2

Pain specialists questioned whether there were any real conceptual differences between intensity and severity, especially when they were talking to CYP about their pain:

I was a little confused about what the difference was meant to be between severity and intensity… from a conceptual point of view...how would you know the difference when you are speaking with a child.

Pain specialist 3

Some pain specialists discussed how the lack of distinction between the 2 concepts meant that they tended to think of them as meaning the same thing. For others, this meant that severity information appeared to be disregarded:

Severity and intensity is very difficult to differentiate between so I’d lose one of those and I think severity is the one I’d lose.

Pain specialist 8

For rheumatology specialists, intensity and severity together were a high priority in their interpretations of pain experiences. Although rheumatology specialists seemed to appreciate similar conceptual issues about considering intensity and severity separately, they mostly talked about both of these concepts interchangeably:

That’s all the information I used...the severity and the intensity

Rheumatology specialist 15

For some people, they’d [severity and intensity] be synonymous, so you ask them the same question twice.

Rheumatology specialist 13

The high prioritization of pain intensity appeared to be unambiguous within the groups despite little explicit discussion around why it was an important feature of interpretation.

##### Theme 2: Prioritization of Pain Location

Pain location information was highly prioritized by both specialist groups. Both focus groups discussed the significance of the number of pain locations in their interpretations, although different discourses regarding this facet of pain were apparent between groups. Pain specialists talked about their prioritizations of the *number of different pain sites*, whereas rheumatology specialists referred to the *number of joints affected*, suggesting rheumatology specialists were linking pain reports with evidence of disease activity, such as inflamed, active, and arthritic joints:

This vignette only had one site so number of sites.

Pain specialist 8

So I kind of took into account how many patients’ joints were involved.

Rheumatology specialist 15

Hot knees, very concise of where the pain is and the number of joints.

Rheumatology specialist 16

Although specific pain in the joints was a high priority for rheumatology specialists, no particular pain location was considered to be significant to pain specialists. Another aspect of pain location information, which was a high prioritization for pain specialists only, was pain spread. Pain spread can relate to how many pain locations there are (as discussed), or how much pain in a specific area radiates to other sites. Pain spread that radiated across several sites appeared to be significant for some pain specialists, with 1 participant suggesting this could be an *over-interpretation*:

So I was over-interpreting that pain because it says elbow but marked her entire arm.

Pain specialist 6

Radiating pain across pain sites was not discussed by rheumatology specialists.

##### Theme 3: Prioritization of Pain Emotion

The biggest contrast in the prioritization of all multidimensional pain information collected was both between and within pain and rheumatology specialist discussions of emotion. For some pain and rheumatology specialists, emotion was a high priority when interpreting CYP pain:

I must admit I did look at the faces so that wasn’t quite so severe perhaps.

Pain specialist 4

I would say I probably ranked using the emotional aspect probably slightly more...So it was more swaying myself towards the emotional impact of that pain.

Rheumatology specialist 11

There seemed to be a number of reasons why pain and rheumatology specialists were cautious about allowing scores on emotion to influence their overall perception of how severe the pain experience was. If emotion did not correspond and was discrepant with the rest of the information that had been given, pain experiences would be ranked lower by pain specialists particularly:

So I might have thought that tears would mean worse and yet it didn’t seem to fit that well with the rest of the information so I ended up disregarding the faces on most of these rather than giving them priority.

Pain specialist 9

Both groups talked about the necessity of additional confirmatory evidence when interpreting emotion scores. Data about function and interference (with medications, mood, and sleep) were important for pain specialists as they sought to weight pain emotion within another concept that they could better understand:

I did somewhat look at the face but I was a little confused what to do with it...because I wasn’t sure why I had only emotion and not function with it, I guess if I had all three (pain, function, and emotion), I might have integrated them.

Pain specialist 6

It’s helpful to know if the child is doing well because of medications they’re on...I always look at pain impact, moods, sleep...you know, interference.

Pain specialist 2

Rheumatology specialists believed that pain emotion should make sense within the context of other clinical observations. This context was necessary because distress could have signified CYP were unhappy with being at clinic, rather than because of the pain they were experiencing:

Because you get some people that are, oh my god, the worst pain I’ve ever had...and you know their heart rates completely normal.

Rheumatology specialist 10

Clearly none of them are happy. So I don't know whether they're not happy because they come to the clinic.

Rheumatology specialist 17

For both pain and rheumatology specialists, the age of CYP reporting pain was important in the interpretation of emotions. The emotions of older children were prioritized higher by both groups of specialists than that of younger children who would more commonly exhibit signs of distress as part of everyday life:

You know 16 year olds, if you’re crying it must really be bad whereas when you are little, that’s just a part of your daily life.

Pain specialist 9

A 16 year old may be better at coping with it, when they’re 14 maybe they’re a bit more emotional.

Rheumatology specialist 16

Rheumatology specialists particularly believed that older children might have more severe emotions associated with their pain because the pain experiences became worse:

It sometimes can be worse for people that are a bit older, they're in constant pain, so it can be difficult.

Rheumatology specialist 10

##### Theme 4: Prioritization of Pain Quality Representations

The use of color in pain reports of CYP was considered to be significant by some pain and rheumatology specialists and insignificant by others. For some, the absence of color in vignette scenarios made for a less powerful depiction of pain:

There wasn’t any use of colour...the visual didn’t seem as powerful to me.

Pain specialist 2

This [particularly ranking the middle vignette] was difficult because there was no colour.

Rheumatology specialist 13

For specialists who did prioritize the use of color in their interpretations of CYP’s pain, use of the color red was high compared with the use of any other colors, as was the intensity of the shading:

Red to me is a colour that children will use when something is bad...there was more red, it felt more intense and more meaningful...Intensity of the colouring, I think there’s something about...the intensity of the shading in.

Pain specialist 8

When they are putting red...for them it’s too painful, red means too painful. And none of them put anywhere in green.

Rheumatology specialist 17

Pain and rheumatology specialists prioritized the use of pain labeling and word descriptors differently within groups. Whereas some viewed labeling as high priority, others placed it low in their interpretations. The vocabulary used was the most significantly prioritized aspect of this feature:

His word labels were sort of middle of the road so he used things like “a little bit.”

Pain specialist 6

I did look at the label a little bit, um thinking about the word “throbbing” for example.

Pain specialist 7

Describing the pain, there is “cracking” painful, I think that was the main reason for ranking this vignette higher.

Rheumatology specialist 14

For some pain and rheumatology specialists, pain symbols were not prioritized highly in their interpretations of pain experiences. Some of the reasons contributing to this were that the meaning of the symbols was confusing, not useful, and too basic for understanding CYP’s actual thought processes regarding pain reporting:

About the symbols, I don’t really use those much either, they actually confuse me a bit.

Pain specialist 4

I think some of the symbols are not useful...If you look through, some have hardly been used.

Rheumatology specialist 13

It doesn’t really give you any impression of what the child is thinking, they’re just very basic symbols.

Rheumatology specialist 18

The only symbol that did seem to hold meaning was the use of the *fire* icon. The number of symbols used also appeared to factor into pain specialist’s interpretation of pain experiences:

If the child’s trying to say the type of pain really the only symbol here that makes sense is the “firey” one...I don’t even know what the rest of them mean.

Pain specialist 6

I didn’t find the symbols very useful except with fire, obviously you know what they feel by fire.

Rheumatology specialist 13

I didn’t pay much attention to which symbol but this vignette put two pictures on it.

Pain specialist 9

There were general overarching differences and commonalities in the ways that the 2 groups prioritized pain quality information. The commonalities for both groups were that prioritization of the color red was high, as was the particular use of the fire symbols in CYP’s pain depictions. The prioritization of use of the color red and fire symbols could again relate to specialists’ focus on disease activity indicators in CYP with JIA.

## Discussion

### Principal Findings

This study is the first to explore the synthesis, salience, and prioritization of multidimensional mHealth pain data from CYP with JIA by key stakeholders involved in long-term pain management. Significant aspects of the researchers’ and HCPs’ subjective scoring systems for interpreting mHealth data were identified through qualitative themes. There were some shared understandings between groups (such as the salience placed upon pain intensity, caution in interpreting pain emotion, and overall low priority of pain quality information) but far more divergent ideas about other features (such as the disregard of pain severity by pain specialists [but not rheumatology specialists], differences in the use of pain location information [importance of sites vs joints], the salience placed upon radiating pain by pain specialists [but not rheumatology specialists], and the type of additional information necessary when interpreting pain emotion and general ambiguity about the prioritization of pain colors, symbols, and labels).

The importance of joint pain for rheumatology specialists indicates the prioritization of disease activity indicators in their pain appraisals. Other findings strengthened the idea that links between pain and disease markers were continuously sought by rheumatology specialists, for example, the attention given to the clinical context when interpreting pain emotion. This presents a challenge because a wealth of research supports the premise that pain levels often do not mirror levels of disease activity [2,4,5]. Presuming that pain and inflammation are equivalent is problematic in this particular group of patients. The pain body manikin adopted in the pain field encourages specialists to quantify pain in terms of body sections [33], whereas in rheumatology, active or limited joints are specified [34]. Quantification of active inflamed joints is a core outcome variable in the assessment of improvement in JIA [35], and our findings demonstrate that rheumatology specialists interpret and affix the salience of pain location information in the context of disease.

Pain emotion was prioritized very differently to other features of multidimensional pain data. This pain facet was only prioritized if other contextual information could be provided (which was not included in the vignette scenarios used in this study), whereas pain location, intensity, and severity were valued independent of any other information. Pain and rheumatology specialists generally disregarded pain emotion because there was no corresponding interference, function or clinical observation data provided for additional context in this study. This suggests that where possible, pain data alongside activity/clinical information should be provided to those managing CYP pain. Following this study, we adapted MPT by adding an assessment of pain interference which appears in MPT after users complete the main pain reporting page.

The reasons why specialists might challenge younger children’s emotion reports could be because they believe that those who are younger are not able to report emotion reliably. Cognitive developmental research suggests that children may differ in the way they explain their emotions [36]; however, the use of emotion-descriptive language has been observed in children as early as 2 years [37]. Vocabulary for pain was important in this study, particularly in the interpretation of pain labels. CYP develop pain vocabularies as young as 18 month and use a select number of words to describe their pain at this age (such as *hurt*, *ow,* and *ouch*) [38]. In younger children, parents and caregivers act as the primary responders, interpreters, and communicators of the children’s pain experiences to HCPs. These dynamic pain communication processes reshape children’s perceptions of pain-specific experiences and emotions over time [14,39]. It could also be that specialists recognize the key roles played by the parents in the reporting of pain-related emotions and therefore recognize the need to interrogate these data to better understand the contextual influences upon pain reporting.

### Comparisons With Previous Work

The importance placed upon pain intensity by pain specialists is not surprising given that intensity is a predominant assessment recommendation by pediatric pain expert groups [40]. Although assessment of pain is mostly neglected in composite outcome measures for JIA, measurement of pain intensity using single-item rating scales occurs in some clinical practices [41]. The conceptual overlap between pain intensity and severity is recognizable from both focus group discussions and from the literature. Operationalization of these key pain terms is almost nonexistent. To our knowledge, only 1 paper discusses key differences between these 2 concepts. Pain intensity has been argued to be defined as how much a patient hurts in quantifiable terms of pain magnitude [42]. However, pain severity is defined as a more global construct that incorporates both intensity and its interference. These data show that professionals attempt to make a distinction between intensity and severity, although the conceptual overlap leads to the disregard of pain severity in some instances and the amalgamation of both intensity and severity in others. The lack of operationalized definitions for professionals is concerning given that CYP are asked to make the same distinction when using pain rating scales [43]. Some specialists in our study were concerned that CYP would choose size of pain based upon the size of the body placement area, rather than to reflect the magnitude of the pain intensity or severity. This emphasizes the need to explore how CYP also denote pain magnitude in multidimensional assessment.

Many of the prioritizations of both pain and rheumatology specialists reflected the focus of professional training and recommendations (for example, rheumatology specialists’ attention to disease activity and clinical context and pain specialists’ attention to interference). For pain specialists, the significance of providing data on function is highlighted in recommendations for the measurement of pediatric chronic pain in clinical trials [40]. Similarly, the significance of clinical contextual factors in rheumatology is apparent throughout core outcome domains for the improvement of JIA [35]. Perceptual set theory is useful in interpreting this finding. This theory posits that the processing of stimuli is actively influenced by a bias or predisposition to pay attention to particular aspects of data, which is usually influenced by individuals’ expectations and culture (including their professional culture) [44,45]. This study indicates that professionals’ interpretations of pain may be influenced by schemata about pain, which have been shaped by specialist training and recommendations. These schemata appear to be guiding the professionals’ different synthetization processes when presented with the same set of pain stimuli.

### Strengths and Limitations

The use of specific stimulus materials in this study encouraged participants to access their own metacognitive thinking (an individual’s awareness of their own cognitive processes [46]) regarding the interpretation of real CYP pain data. ‘Focus group discussions may have been superficial without stimulus prompts to encourage discussions, and participants may have been less likely to talk in-depth about the processes behind their interpretations. Group discussions lead to validation and extension of individual participants’ accounts, which is important when the topic area is this complex [47].

A limitation of this study concerns the participants working in different health care systems, which may have influenced their interpretations of pain data. Many of the findings of this study can be related to professional measurement recommendations that are country specific. The pain specialist group was predominantly based in Canada and the United States, whereas the rheumatology specialist group worked in the United Kingdom.

The data collection methods used within this study also had some limitations. The setting for the focus groups in which data were collected may have added extra pressure to the participants whose attention and time were already stretched because of their conference attendance. This may have had an impact on the breadth and depth of issues explored. However, participants had received the vignettes before the meeting, which ensured that they had additional time to reflect on the issues. In addition, the contributors were all professionals and were therefore accustomed to expressing their views in time-limited contexts.

### Future Research

These findings could be used to inform the ways in which pain information is best presented to key stakeholders in pain management in the future. Data presented could emphasize only informational features that are high priority, could reduce the burden of certain decision-making processes where information can automatically be combined, or could take away any redundant informational features. Information visualizations are useful for representing rich, abstract, and complex information by simulating people’s natural perceptual processes in a more efficient manner [48]. To develop these, exploiting and capturing pain interpretation processes based upon group, rather than individual, decision-making processes is necessary for creating less subjective systems of interpretation. However, in our other study developing MPT, we found that CYP want to be able to communicate all of the components of pain as featured in the app. If we reduce the amount of burden on interpreters, we risk detracting from the context and nature of these types of assessments. Multidimensional tools are advantageous in that they encourage discussion and communication about pain, which is important to continue to accommodate in practice, even where professionals might only use specific parts of pain information to inform their management decisions.

### Conclusions

To our knowledge, this study is the first to focus on the processes of interpreting mHealth multidimensional pain data rather than on the development of such tools, which is important given the increase in the use of mHealth technology in the management of pediatric chronic conditions [23]. The conceptual framework of pain assessment and interpretation is complex. These findings are important for the development of interpretive guidelines for new pain assessment tools that aim to capture complex data. Particularly, these findings are useful for future research that aims to develop appropriate pain data visualizations that are useful to key stakeholders managing pain in clinics and research.

## Multimedia Appendix 1

Vignette scenarios provided.

## Multimedia Appendix 2

Consolidated Criteria for Reporting Qualitative Research checklist.

## Abbreviations

CYP: children and young people
HCP: health care professional
JIA: juvenile idiopathic arthritis
mHealth: mobile health
MPT: My Pain Tracker
NHS: National Health Service
NIHR: National Institute for Health Research
