# Supplementary material for: “Seeing Pain Differently”: A Qualitative Investigation Into the Differences and Similarities of Pain and Rheumatology Specialists’ Interpretation of Multidimensional Mobile Health Pain Data From Children and Young People With Juvenile Idiopathic Arthritis
Source: JMIR Mhealth Uhealth. 2019 Jul 2;7(7):e12952. doi: 10.2196/12952 (PMC6632104; doi:10.2196/12952)
Supplement: Supplementary file 1 [file mhealth_v7i7e12952_app1.pdf]

Vignette 1 - Tom

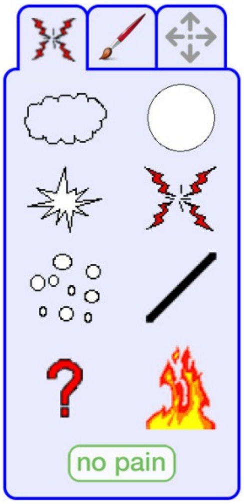

Age 14

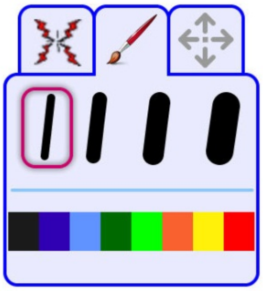

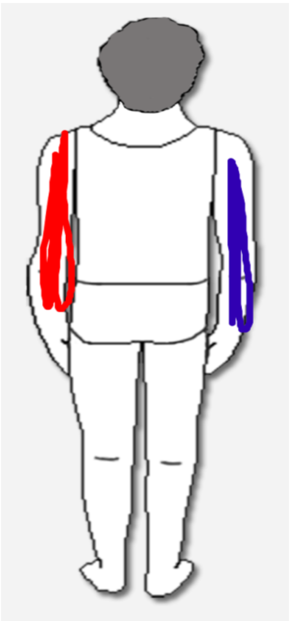

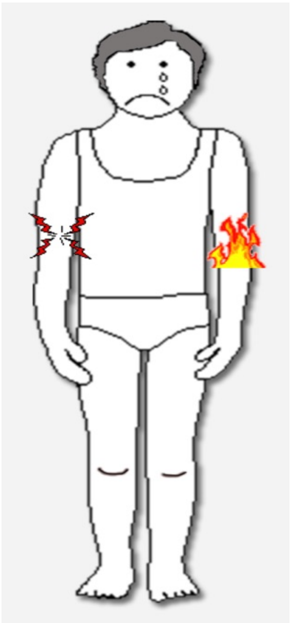

Emotions Labelled

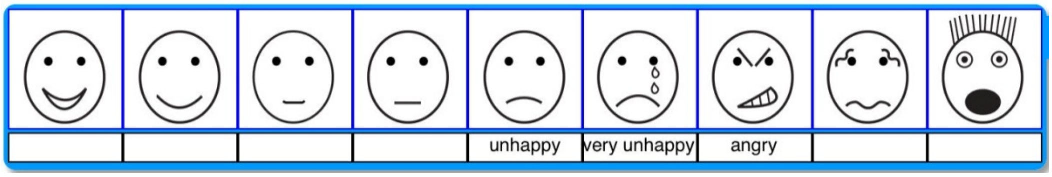

- Chosen Emotion Label: *Very unhappy* 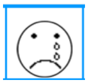 Face number 6

Location (Joints)	Symbol	Severity<br>1 - 5	Intensity<br>1 - 5	Associated body map area (MCR)	Colour	label
**Right elbow**	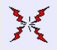	4	3	2	blue	
				1	blue	
				3	blue	
**Left elbow**	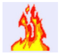	3	3	6	red	Hot pain
				5	red	Heat hotter
				7	red	Heat hotter

Vignette 2 -Samantha

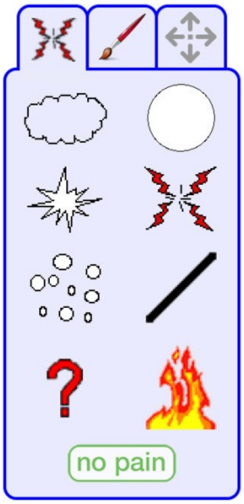

Age 12

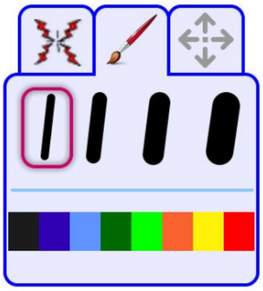

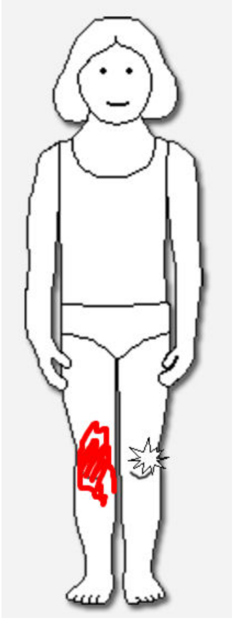

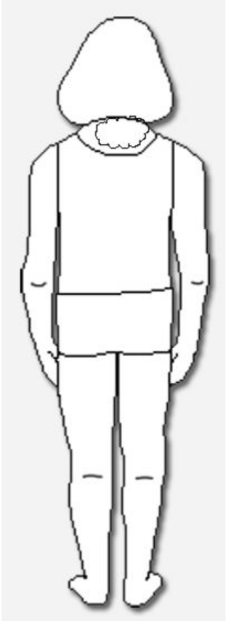

Emotions Labelled:

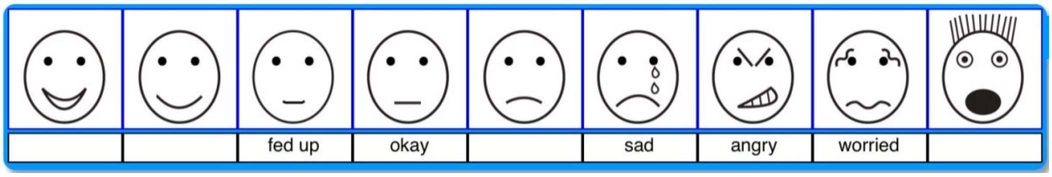

- Chosen Emotion Label: *Fed up* 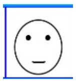 Face number 3

Location (Joints)	Symbol 1	Severity 1- 5	Intensity 1-5	Associated body map area (MCR)	Colour	label
**Left Knee**	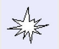	3	2	14		Nagging
**Neck**	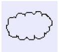	3	2	24		Sore
**Right Knee**				9	Red	
				10	Red	
				11	Red	

Vignette 3 - Anna

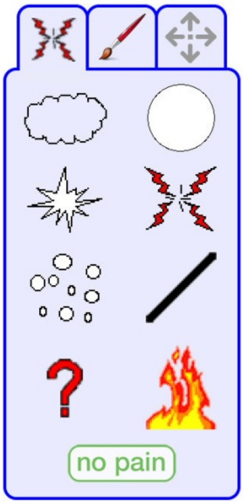

Age 14

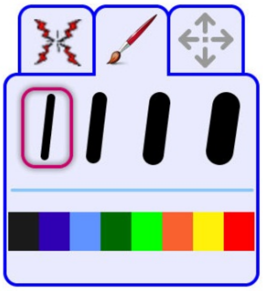

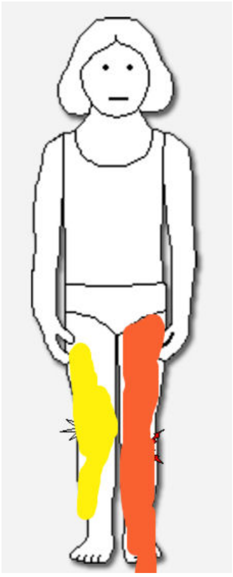

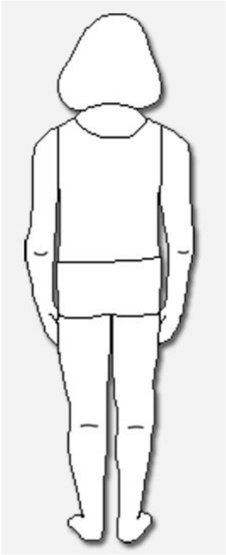

Emotions Labelled

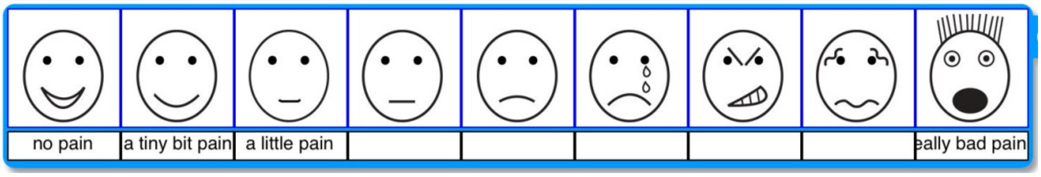

- Chosen Emotion Label: no label 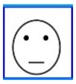 Face number 4

Location (Joints)	Symbol 1	Severity 1 -5	Intensity 1 -5	Associated body map area (MCR)	Colour	label
**Right Knee**		3	4	10	Yellow	A little bit of pain
				9	Yellow	
				11	Yellow	
**Left Knee**		1	2	14	Orange	Small pain
				13	Orange	
				15	Orange	
				16	Orange	

Vignette 4 – Nicola

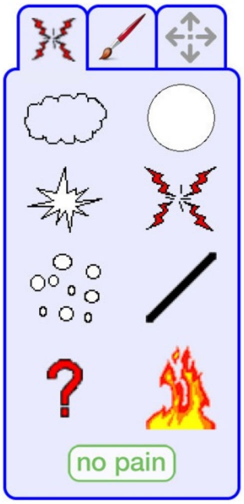

Age 16

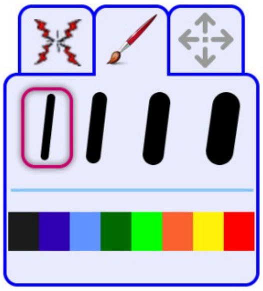

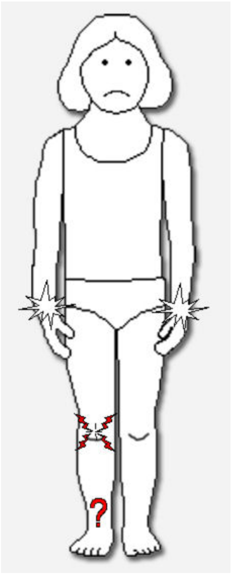

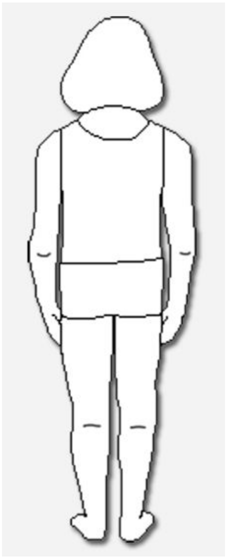

Emotions Labelled

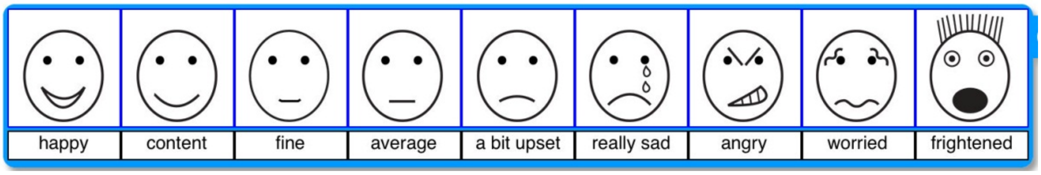

- Chosen Emotion Label: a bit upset 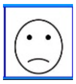 Face number 5

Location (Joints)	Symbol 1	Severity 1 -5	Intensity 1 -5	Associated body map area (MCR)	Colour	label
**Right wrist**	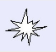	4	1	3		Constantly sore
				4		Constantly sore
**Left wrist**	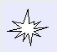	4	1	7		Sore
				8		Sore
**Right Knee**	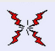	2	1	10		Sore/achy
**Right Ankle**	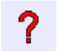	2	3	11		Progressively sore after walking
				12		Progressively sore after walking

Vignette 5 -Rose

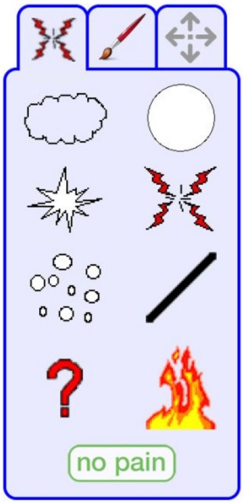

Age 9

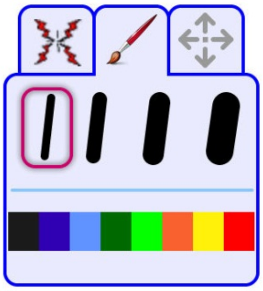

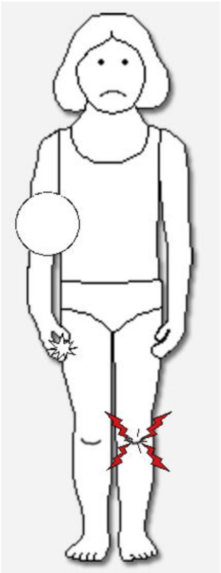

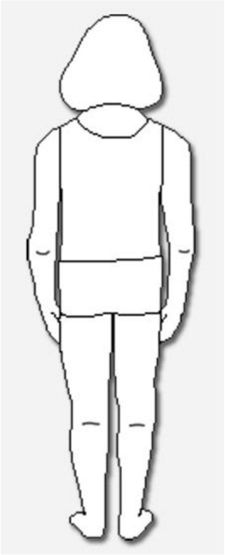

Emotions Labelled

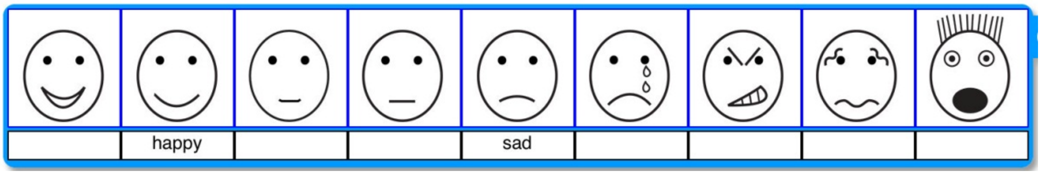

- Chosen Emotion Label: Sad 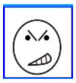 Face number 5

Location (Joints)	Symbol 1	Severity 1 -5	Intensity 1 -5	Associated body map area (MCR)	Colour	label
**Right elbow**	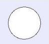	5	3	2		hurting
**Right metacarpophalangeal (Finger)**	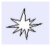	1	3	4		
**Left Knee**	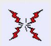	5	3	14		

Vignette 6 - Clark

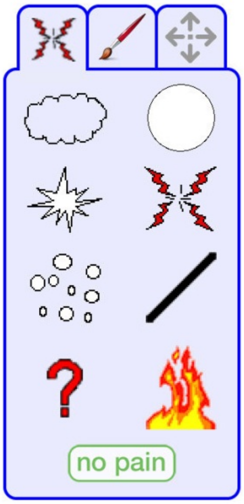

Age 12

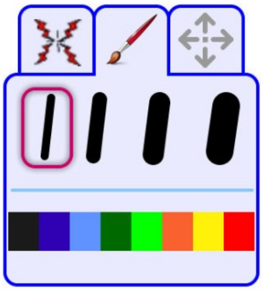

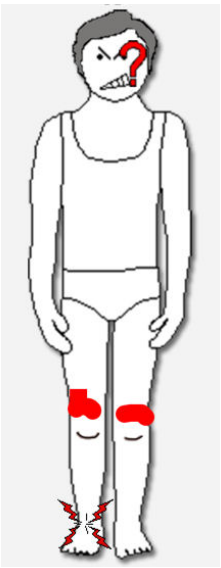

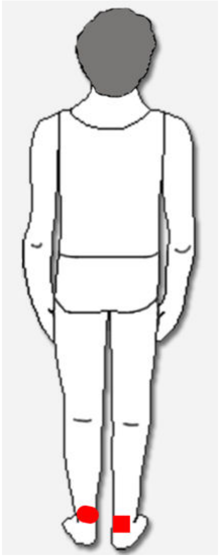

Emotions Labelled

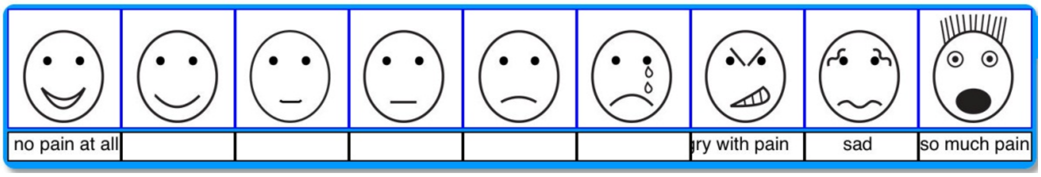

- Chosen Emotion Label: Angry with pain 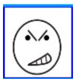 Face number 7

Location (Joints)	Symbol 1	Severity 1 -5	Intensity 1 -5	Associated body map area (MCR)	Colour	label
Left TMJ (jaw)	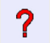	5	3	17		
Right ankle	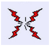	4	5	12	Red	Throbbing
Left Ankle				16	Red	
Left Knee				14	Red	
Right Knee				10	Red	

Vignette 7 - Ben

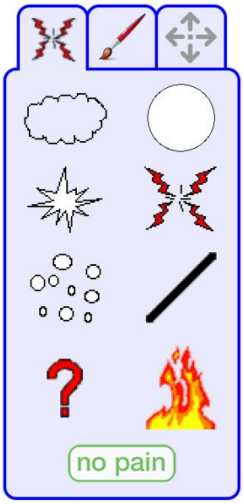

Age 16

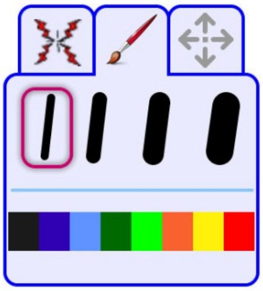

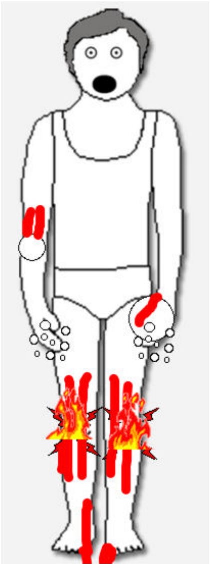

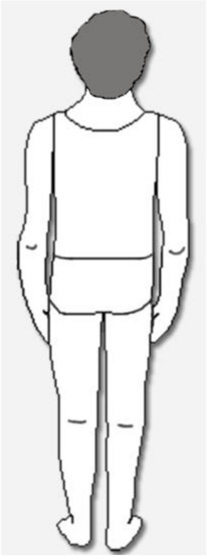

Emotions Labelled

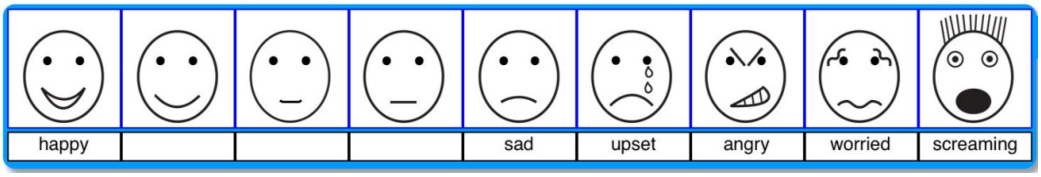

- Chosen Emotion Label: screaming 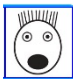 Face number 9

Location (Joints)	Symbol 1	Severity 1 -5	Intensity 1 -5	Symbol 2	Severity 1 -5	Intensity 1 -5	Associated body map area (MCR)	Colour	label
**Right elbow**	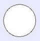	1	3				2	Red	stretching pain
							1	Red	
**3 left MCP**	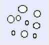	2	3	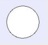	3	3	8	Red	Thumb bone out of place
**Left knee**	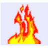	3	3	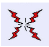	4	4	14	Red	
							13	Red	
							15	Red	
**Right knee**	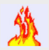	3	3	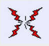	4	4	10	Red	Cracks & painful
							9	Red	
							11	Red	
**right MPC**	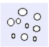	2	3				4		

Vignette 8 - Penny

Age 8

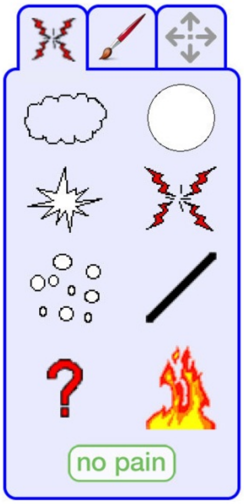

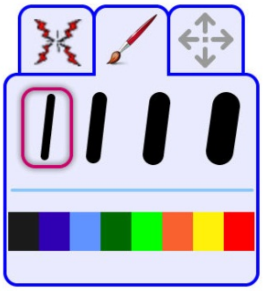

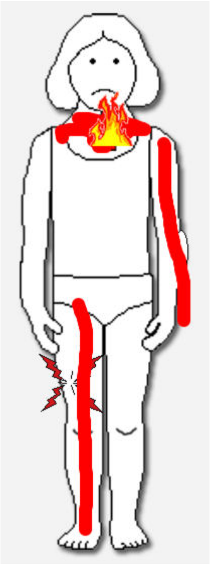

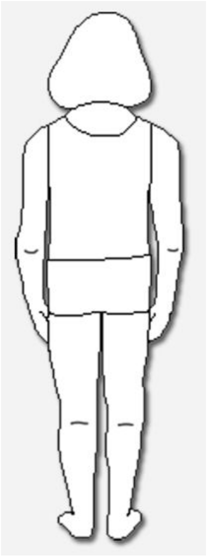

Emotions Labelled

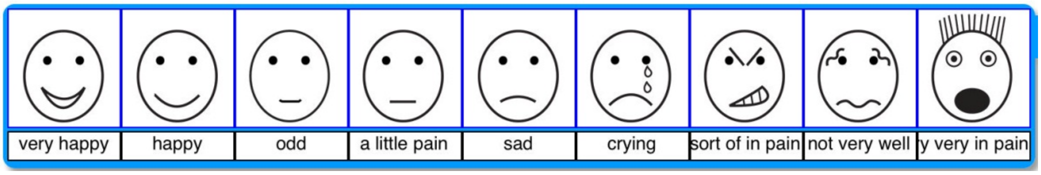

- Chosen Emotion Label: Sad 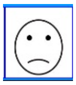 Face number 5

Location (Joints)	Symbol 1	Severity 1 -5	Intensity 1 -5	Symbol 2	Severity 1 -5	Intensity 1 -5	Associated body map area (MCR)	Colour	label
**Streno-clavicular (Chest)**	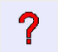	5	3	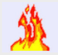	4	5	1	Red	Throat hurts
							5	Red	Throat burns
							18	Red	
**Left elbow**	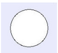	2	4				6	Red	Makes me sad
							5	Red	
							7	Red	
**Right Knee**	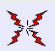	5	5				10	Red	
							9	Red	
							11	Red	
							12	Red	

Vignette 9 – Aaron

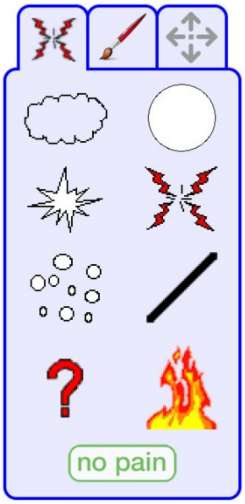

Age 9

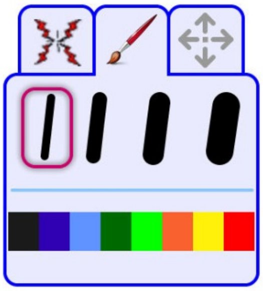

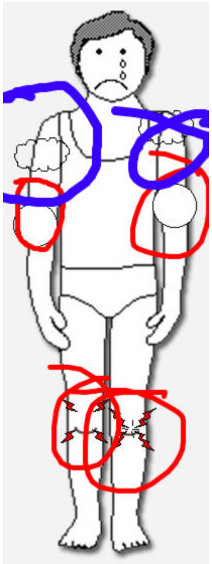

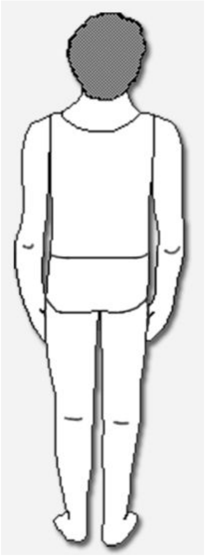

Emotions Labelled:

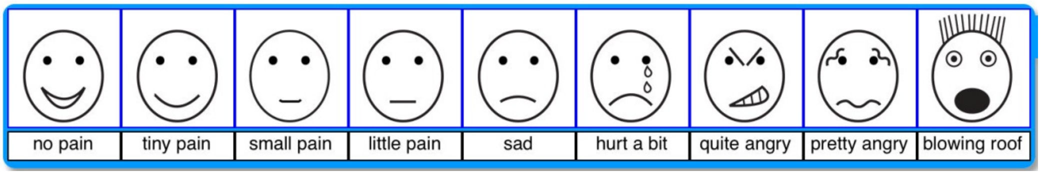

- Chosen Emotion Label: Hurt a bit 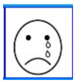 Face number 9

Location (Joints)	Symbol 1	Severity 1 -5	Intensity 1 -5	Associated body map area (MCR)	Colour	label
**Right shoulder**	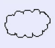	4	1	1	blue	A little
**Right elbow**	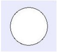	2	3	2	Red	Cracks, stretching
				1	Red	
				3	Red	
**Left shoulder**	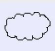	4	1	5	Blue	A little
**Left elbow**	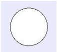	4	4	6	Red	
				5	Red	
				7	Red	
**Left Knee**	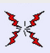	3	3	14	Red	A bit
**Right knee**	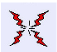	3	3	10	Red	A bit
