# Supplementary material for: “Seeing Pain Differently”: A Qualitative Investigation Into the Differences and Similarities of Pain and Rheumatology Specialists’ Interpretation of Multidimensional Mobile Health Pain Data From Children and Young People With Juvenile Idiopathic Arthritis
Source: JMIR Mhealth Uhealth. 2019 Jul 2;7(7):e12952. doi: 10.2196/12952 (PMC6632104; doi:10.2196/12952)
Supplement: Supplementary file 2 [file mhealth_v7i7e12952_app2.pdf]

**Multimedia appendix 1- COREQ Checklist**

| Consolidated criteria for reporting qualitative studies (COREQ): 32-item checklist. | | | |
|---|---|---|---|
| No. | Item | Guide questions/description | Response/page number referenced |
| Domain 1: Research team and reflectivity | | | |
| Personal characteristics | | | |
| 1. | Interviewer/facilitator | Which author/s conducted the interview or focus group? | Page 6 (Procedure section): "Participants attending the focus groups were given a standardised presentation by the study team (A.R., D.G. and R.R.L)" |
| 2. | Credentials | What were the researcher's credentials? E.g. PhD, MD | Page 6 (Procedure section): " At the time of data collection, A.R. was a Research Associate (trained to PhD level in Psychology and Medical research) and D.G. and R.R.L. were PhD students (trained to MSc level in Health Psychology and training in Psychology and Medical research)." |
| 3. | Occupation | What was their occupation at the time of the study? | Page 6 (Procedure section, as above). |
| 4. | Gender | Was the researcher male or female? | Not relevant in the context of this study as would not have affected conduct or analysis of focus groups on pain assessment (Male and female researchers involved). |
| 5. | Experience and training | What experience or training did the researcher have? | Page 6 (Procedure section): "All researchers involved in data collection and analysis had conducted and been involved in prior qualitative research studies and were closely supervised in preparing and conducting focus groups by L.C. (Senior Lecturer and practitioner in Health Psychology)." |
| Relationship with participants | | | |
| 6. | Relationship established | Was a relationship established prior to study commencement? | Not applicable as professional relationships were used as the basis for recruitment (Purposive [based on interests] and snowballing). Inherent in the methodology stated is evidence of some existing professional relations. |
| 7. | Participant knowledge of the interviewer | What did the participants know about the researcher? e.g. personal goals, reasons for doing the research. | Page 5 (Sample and recruitment section): "Selected participants were emailed with the aims of the study and briefed about what would be involved. |

| | | | |
|---|---|---|---|
| | | | Participants were also given a brief background to the rationale of the study, the research group's work and who the researchers involved on the study were." |
| 8. | Interviewer characteristics | What characteristics were reported about the interviewer/facilitator? e.g. Bias, assumptions, reasons and interests in the research topic. | Bias/assumptions reported on Page 6 (Procedure section):<br>"The researchers conducting this study did not have particular experience of managing pain either in a pain or rheumatology focused medical context."<br><br>Reasons and interest in the topic area reported on Page 4 (Introduction section):<br>"Recent research on the implementation of multi-dimensional paediatric pain assessment tools has primarily focused on development [17;18], rather than on the utilisation and interpretation of information from such measures." |
| Domain 2: study design | | | |
| Theoretical framework | | | |
| 9. | Methodological orientation and theory | What methodological orientation was stated to underpin the study? e.g. grounded theory, discourse analysis, ethnography, phenomenology, content analysis | Page 4 (Study design):<br>"We incorporated aspects of Q-methodology research into the focus groups and presented real mHealth multi-dimensional pain data from CYP with JIA to participants."<br><br>Page 7 (Data collection):<br>" Focus group data were analysed using a step-by-step guide for conducting deductive latent thematic analysis [25]." |
| Participant selection | | | |
| 10. | Sampling | How were participants selected? e.g. purposive, convenience, consecutive, snowball | Page 5 (Sample and recruitment):<br>" Two groups of participants recruited through purposive sampling took part in the study"<br>" Interested participants responded directly to the authors and were encouraged to send the email to colleagues with similar interests/specialisations (snowball sampling [21])." |

| | | | |
|---|---|---|---|
| 11. | Method of approach | How were participants approached? e.g. face-to-face, telephone, mail, email | Page 5 (Sample and recruitment):<br>" Selected participants were emailed with the aims of the study and briefed about what would be involved." |
| 12. | Sample size | How many participants were in the study? | Page 7 (Participant characteristics):<br>" Nineteen participants took part in the focus groups; Nine pain specialists in one focus group (Participants 1-9) and ten rheumatology specialists (Participants 10-19) in the other focus group" |
| 13. | Non-participation | How many people refused to participate or dropped out? Reasons? | Not applicable- All who registered interest took part in the focus groups. |
| Setting | | | |
| 14. | Setting of data collection | Where was the data collected? e.g. home, clinic, workplace | Page 5 (Setting):<br>" Two separate focus groups were conducted, with the pain specialist focus group held at an international pain conference (USA) and the rheumatology specialist focus group held at a national rheumatology conference (UK)." |
| 15. | Presence of non-participants | Was anyone else present besides the participants and researchers? | Not applicable- no other persons present during conduct of focus groups. |
| 16. | Description of sample | What are the important characteristics of the sample? e.g. demographic data, date | Page 7 (Participant characteristics):<br>Table 1. |
| Data collection | | | |
| 17. | Interview guide | Were questions, prompts, guides provided by the authors? Was it pilot tested? | Not applicable as no specific interview guide however stimuli (Vignette scenarios) used to prompt focus group discussions are provided in Multimedia appendix 2. |
| 18. | Repeat interviews | Were repeat interviews carried out? If yes, how many? | Not applicable- interviews not conducted. |
| 19. | Audio/visual recording | Did the research use audio or visual recording to collect the data? | Page 6 (Procedure section):<br>"Focus group discussions provided qualitative data for analysis and were audio-recorded and transcribed verbatim for analysis." |
| 20. | Field notes | Were field notes made | Page 7 (Procedure section): |

| | | | |
|---|---|---|---|
| | | during and/or after the interview or focus group? | "Field notes collected by the researchers during the conduct of focus groups were used to provide additional context to the analytical process." |
| 21. | Duration | What was the duration of the interviews or focus group? | Page 7 (Participant characeristics):<br>" The pain specialist focus group lasted for approximately 48 minutes and the rheumatology specialist focus group ran for approximately 75 minutes." |
| 22. | Data saturation | Was data saturation discussed? | Not applicable as not an aim of this particular study. |
| 23. | Transcripts returned | Were transcripts returned to participants for comment and/or correction? | Not applicable as transcripts were not returned to participants. |
| Domain 3: analysis and findings | | | |
| Data analysis | | | |
| 24. | Number of data coders | How many data coders coded the data? | Page 7 (Data analysis):<br>" Focus group data were analysed using a step-by-step guide for conducting deductive latent thematic analysis by R.R.L and L.C [25]." |
| 25. | Description of the coding tree | Did authors provide a description of the coding tree? | Page 9 and 10 (Figure 2) shows coding trees for all themes identified. |
| 26. | Derivation of themes | Were themes identified in advance or derived from the data? | Page 7 (Data analysis):<br>"Coders defined what would be considered a significant theme prior to data analysis (issues about pain prioritisation and interpretation of pain features, in line with the aims of the study)". |
| 27. | Software | What software, if applicable, was used to manage the data? | Page 6 (Procedure section):<br>"All audio-recorded interviews were uploaded to and analysed in NVivo 10 (QSR International, Doncaster, Australia)." |
| 28. | Participant checking | Did participants provide feedback on the findings? | Not applicable as participants did not provide feedback on the data/findings. |
| Reporting | | | |
| 29. | Quotations presented | Were participant quotations presented to illustrate the themes / findings? Was each quotation identified? e.g. participant number. | Page 11-Page 14 (Results section):<br>Quotations are presented throughout the text alongside interpretations. |
| 30. | Data and findings consistent | Was there consistency between the data presented and the | Page 11-14 (Results section): Quotations are presented alongside interpretations for transparency between data and |

| | | | |
|---|---|---|---|
| | | findings? | findings. |
| 31. | Clarity of major themes | Were major themes clearly presented in the findings? | Page 11-14 (Results section):<br>Major themes are presented which relate to the pain features identified by My Pain Tracker (and as identified earlier in the data analysis section, major themes relate to a priori themes expected). |
| 32. | Clarity of minor themes | Is there a description of diverse cases or discussion of minor themes? | Page 11-14 (Results section):<br>Minor themes are presented in thematic diagrams (Figure 2) and discussed in more detail through quotations and interpretations under major theme headings. |
